# Technetium Complexes and Radiopharmaceuticals with Scorpionate Ligands

**DOI:** 10.3390/molecules23082039

**Published:** 2018-08-15

**Authors:** Petra Martini, Micol Pasquali, Alessandra Boschi, Licia Uccelli, Melchiore Giganti, Adriano Duatti

**Affiliations:** 1 Department of Morphology, Surgery and Experimental Medicine, University of Ferrara, 44121 Ferrara, Italy; mrtptr1@unife.it (P.M.); psqmcl@unife.it (M.P.); bsclsn@unife.it (A.B.); ccl@uinfe.it (L.U.); ggm@unife.it (M.G.); 2 Department of Chemical and Pharmaceutical Sciences, University of Ferrara, 44121 Ferrara, Italy

**Keywords:** scorpionate ligands, technetium, Tc cores, Tc-99m radiopharmaceuticals

## Abstract

Scorpionate ligands have played a crucial role in the development of technetium chemistry and, recently, they have also fueled important advancements in the discovery of novel diagnostic imaging agents based on the γ-emitting radionuclide technetium-99m. The purpose of this short review is to provide an illustration of the most general and relevant results in this field, however without being concerned with the details of the analytical features of the various compounds. Thus, emphasis will be given to the description of the general features of technetium complexes with scorpionate ligands including coordination modes, structural properties and an elementary bonding description. Similarly, the most relevant examples of technetium-99m radiopharmaceuticals derived from scorpionate ligands and their potential interest for nuclear imaging will be summarized.

## 1. Introduction

**Figure 1:**
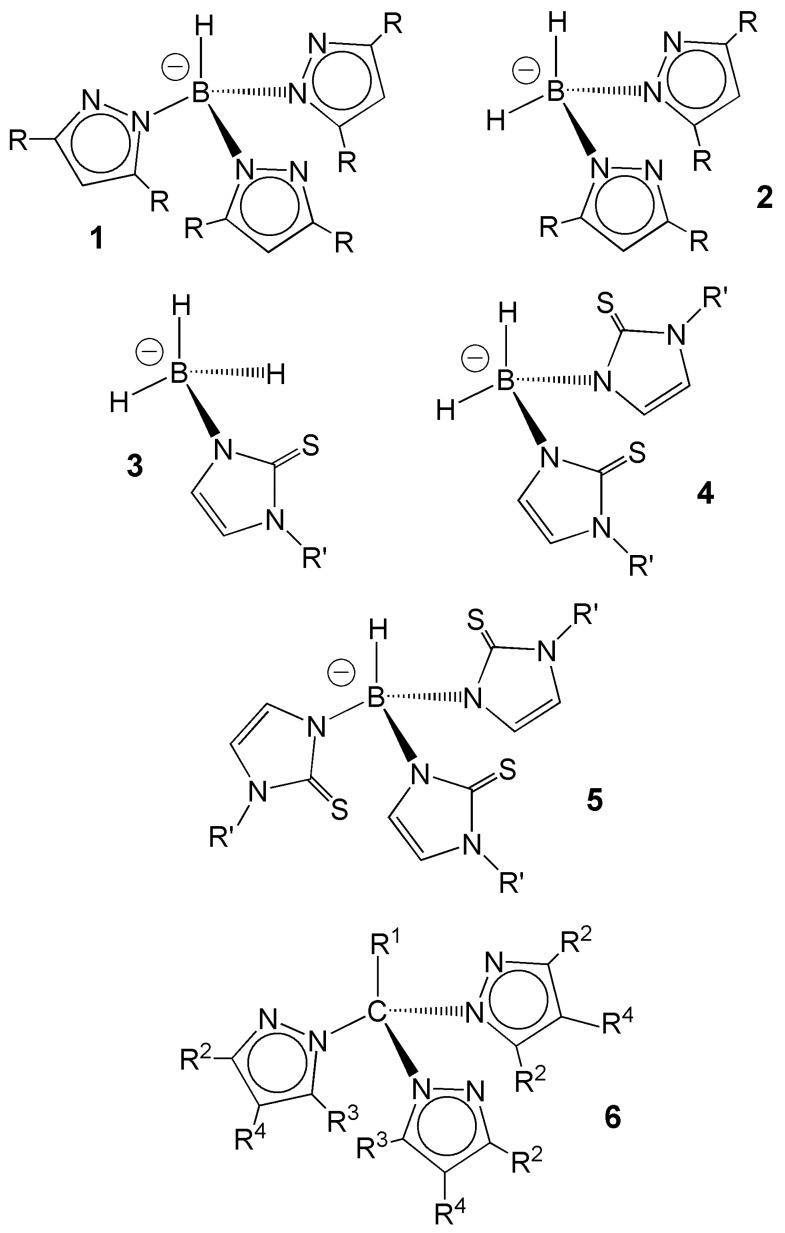
Schematic drawings of the chemical structure of scorpionate ligands. Poly(pyrazolyl)borates, [HB(R_2_pz)_3_]^−^ (**1**,**2**), poly(mercaptoimidazolyl)borates [HB(Rmz)_3_]^−^ (**3**), [HB(Rmz)_2_^−^ (**4**), [HB(Rmz)]^−^ (**5**), tris(pyrazolyl)methane [HC(R_3_pz)_3_] (**6**), R, R’, R^1,2,3,4^ = organic functional groups.

Tridentate *N*,*N*,*N*-donor ligands hydrotris(pyrazolyl)borates and tris(pyrazolyl)methanes form a class of versatile tripodal ligands combining a central, tetrahedral boron atom with a variable number (*n* = 2–4) of azolyl rings. These ligands are widely recognized under the suggestive name of ‘scorpionates’ [1,2,3,4,5] that originates from their peculiar coordination mode. Pictorially, a sorpionate ligand can grab a metal ion through the three pyrazolyl rings like the pincers and tail of a scorpion. Since their first introduction these coordinating agents have been used as anchoring ligands to stabilize technetium complexes in a variety of oxidation states from +7 to +1 [4,5]. Similarly, *S*,*S*,*S*-donor ligands poly(mercaptoimidazolyl)borates, which can be viewed as soft congeners of the classical poly(pyrazolyl)borates, have been extensively investigated in technetium coordination chemistry [6,7,8]. Most importantly, due to the fundamental role played by the nuclear isomer technetium-99m in diagnostic nuclear medicine, these ligands have been widely studied for the development of new diagnostic radiopharmaceuticals as imaging agents for different biological targets. An illustration of the chemical structures of the scorpionate ligands discussed in this review is reported in Figure 1.

In this respect, major recognition should be given to the research group of Santos at the Technological and Nuclear Institute in Lisbon, which carried out a multitude of excellent studies in this field, particularly in the synthesis of scorpionate complexes with technetium in low oxidation states [5,9,10]. The same group has been able to translate a number of these inorganic complexes to ^99m^Tc radiopharmaceuticals that exhibited interesting biological properties for their potential use as diagnostic agents. Extensive reports of this research activity, and of other important contributions to the field, have been described in many review articles that should be consulted for obtaining a broad overview of technetium scorpionate chemistry and radiopharmaceuticals [9].

In conventional Single Photon Emission Computed Tomography (SPECT) imaging ^99m^Tc is still the most widely employed radioisotope. This prominent position stems from its very favorable nuclear properties, which includes the emission of nearly monochromatic γ photons with energy of 140 keV, a half-life of 6.06 h allowing easy preparation and administration of ^99m^Tc radiopharmaceuticals, and its ready availability at low costs from the transportable ^99^Mo/^99m^Tc generator [10,11]. The last decades have witnessed a progressive switch of the investigation from SPECT tracers to Positron Emission Tomography (PET) radiopharmaceuticals. This change was mostly driven by the superior imaging properties of PET as compared to SPECT. More precisely, PET is characterized by a higher spatial resolution and sensitivity than conventional SPECT that makes it more accurate in detecting small lesions. However, this scenario is expected to change rapidly after the introduction into the clinical practice of new advanced SPECT cameras offering unprecedented imaging characteristics [12,13]. In particular, these new SPECT technologies significantly narrow the gap in sensitivity between the two nuclear imaging modalities and, interestingly, outperform PET in showing a much higher spatial resolution that is gradually approaching the one-millimeter scale. It is worthy to remind here that PET has an intrinsic physical limit in spatial resolution because of the peculiar features of the positron annihilation process that does not exactly occur at the point of emission of the positron and is energy dependent. Another favorable characteristic of the new SPECT cameras is that they allow collecting tomographic images only in a few min whereas a PET scan usually takes more than 10 min to be completed.

Boosted by these new technological advancements, it is reasonable to expect a renovated interest in ^99m^Tc radiopharmaceuticals and, possibly, a reconsideration of the potential clinical utility of ^99m^Tc tracers that have been set aside because of the past emphasis on PET agents. In this short review, an illustration of the most important classes of technetium scorpionate complexes will be overviewed with special focus on those compounds that have been also prepared at no-carrier added level with the radioisotope ^99m^Tc.

## 2. Technetium Cores

**Figure 2:**
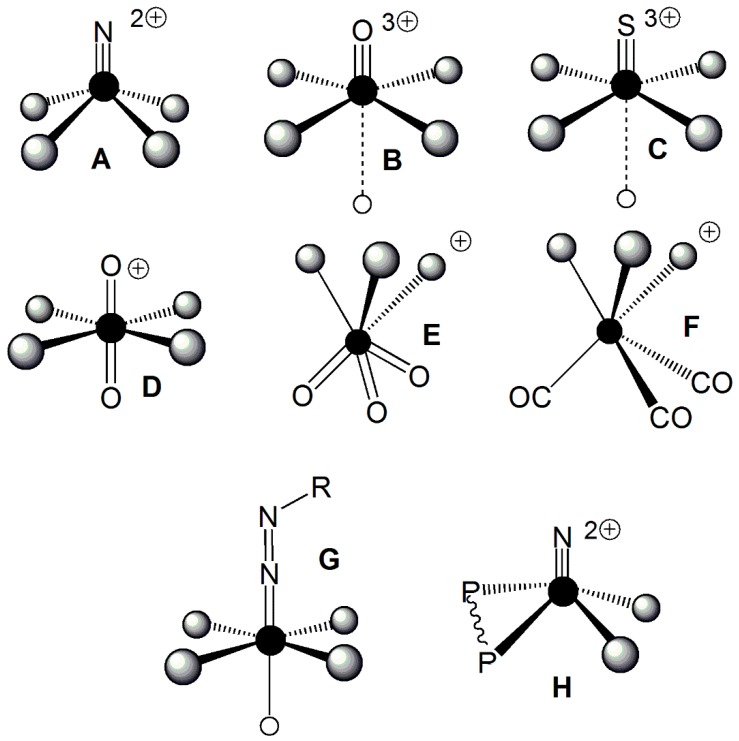
The most important technetium cores. Tc-nitrido (**A**); Tc-oxo (**B**); Tc-sulfido (**C**); Tc-dioxo (**D**); Tc-trioxo (**E**); Tc-*tris*-carbonyl (**F**); Tc-HYNIC (**G**); Tc-nitrido-diphosphane (**H**).

The chemistry of the element technetium is elegantly rich as it is shaped by a multitude of different molecular geometries and oxidation states. In particular, the versatile chemical reactivity of this transition metal can generate characteristics chemical motifs that have been called ‘cores’, ‘metallic functional groups’ or ‘metallic fragments’ [10,11]. These chemicals entities are usually formed by a technetium atom, in a specific oxidation state, combined with some characteristic ligand or set of ligands. Essentially, two main categories of technetium cores can be defined. In the first category, technetium is coordinated to a single atom or group of atoms that can exist only when tightly bound to the metal ion. In the other category, a single ligand, or a set of identical ligands, that can freely exist also when not linked to the metal, strongly bound the technetium ion, thus forming a highly stable arrangement that, upon further reaction with other ligands, is always preserved and remains chemically unaltered. Relevant examples of the first type of technetium functional moieties are the Tc-trioxo ([*mer*-TcO_3_]^+^) [14,15] Tc-oxo ([Tc≡O]^3+^) [10,11], Tc-sulfido ([Tc≡S]^3+^) [16], Tc-nitrido [Tc≡N]^2+^ [17,18] and Tc-dioxo (*trans*-[O=Tc=O]^+^] [10,11] cores. The most important examples of the second type of technetium moieties are the Tc-*tris*-carbonyl (*fac*-[Tc(CO)_3_]^+^) [19,20] and Tc-HYNIC ([Tc=N–NH–C_5_H_5_N] [21] metallic fragments. A unique example of functional group where the two types of cores are intimately entangled into the same moiety is provided by the mixed Tc-nitrido-diphosphane ([(PNP)Tc≡N]^2+^) metallic fragment [22]. Chemical drawings of these functional groups are illustrated in Figure 2.

## 3. Qualitative Analysis of the Bonding of Scorpionate Ligands to Tc Cores

**Figure 3:**
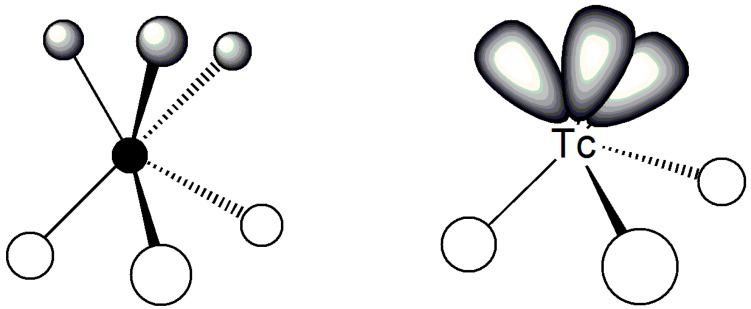
Simplified pictorial representation of the *C_3v_* geometry and of the frontier σ-orbitals for the Tc-trioxo and Tc-tricarbonyl metallic fragments.

Most of the so-called homoscorpionates are nitrogen donor ligands where nitrogen atoms are incorporated in the aromatic system of a pyrazole ring. Although advanced theoretical analyses of the bonding between scorpionate ligands and metal atoms have been reported [23], for the purpose of this review a basic orbital picture could be useful to get some essential insight into the most relevant structural features of the interaction of these ligands with technetium cores. In this elementary picture, pyrazolyl nitrogen atoms can be considered as possessing *sp*^2^ hybridization where one *s*- and two *p*-type atomic orbitals are involved in the σ bonding within the pyrazole ring, whereas the remaining *p*-type orbital perpendicular to the ring plane is involved in the delocalized aromatic π–bonding. Since only one of the two nitrogen atoms forms three σ bonds with aromatic carbon and boron atoms, whereas the other forms only two σ bonds, this latter atom is left with a fully occupied *sp*^2^ σ-orbital that nicely account for its σ-donor ability. It turns out that technetium metallic fragments possessing empty σ-acceptor frontier orbitals are ideal substrates for bond formation with scorpionate ligands (Figure 3).

The other fundamental family of scorpionate ligands is composed by derivatives where the pyrazole ring is replaced by a different heterocycle, thus forming the so-called heteroscorpionates. The most relevant ligands of these type for technetium chemistry and its radiopharmaceuticals is when the aromatic ring is substituted by a 2-mercaptoimidazole moiety (Figure 1). Poly-(mercaptoimidazolyl)borate ligands are considered softer than their poly(pyrazolyl)borate congeners because they coordinate the metal through the soft thione sulfur atom. This heteroatom lies outside the aromatic ring, but assuming it is described as *sp* hybridized, it should possess one filled σ-donor orbital isolobal to that of the nitrogen atom in hard scorpionates. As a result, these soft scorpionates display a coordination ability for transition metals similar to their harder counterparts. In particular, tris(mercaptoimidazolyl)borates act as tripodal ligands and usually span three positions on the triangular face of the coordination polyhedron. However, there exist a few remarkable differences between these two categories of ligands. The supposed *sp* hybridization of the thione sulfur atom leaves one filled *p* orbital available for π-bonding with the metallic group. Notably, this possibility is neatly favored by the fact that although tris(pyrazolyl)borate forms complexes with six-membered chelate rings, *tris*(mercaptopyrazolyl)borates give rise to eight-membered chelate rings, because the sulfur donor atom is positioned out the aromatic ring. This allows rotation of the mercaptoimidazolyl ring around the B–N bond that, in contrast, is strongly hindered for the pyrazolyl ring. This higher rotational freedom may increase the number of accessible spatial orientations potentially suitable for π-orbital overlap between the metallic core and the thioimidazolyl moiety.

Another crucial parameter to be considered in this elementary analysis of the bonding between technetium cores and scorpionates is the symmetry of the final complexes. It is well-known that tripodal ligands impart a *C_3v_* symmetry to the metal center. Thus, technetium cores able to acquire a *C_3v_* arrangement are highly favored in forming stable complexes with tripodal scorpionates. This consideration rules out the Tc-nitrido core since this moiety exclusively prefers a *D_4h_* umbrella-shaped geometry and, consequently, does not exhibit the required three σ-acceptor frontier orbitals positioned in a *fac* scaffold. Conversely, the Tc-trioxo and Tc-tricarbonyl cores do appear as almost ideal substrates for accommodating a tripodal σ-donor ligand as their frontier orbitals contains exactly three σ-acceptor orbitals in a *C_3v_* symmetry (Figure 3). The Tc-oxo and Tc-sulfido cores display an intermediate behavior. Despite their similarities with the Tc-nitrido functional group, the Tc≡O and Tc≡S bond distances are longer than that of the Tc≡N bond. This results from the much stronger π-donor character of the nitrido ([N]^3−^) group as compared to the oxo ([O]^2−^) and sulfido ([S^2−^) groups. A weaker π-donor character implies that the Tc-oxo and Tc-sulfido cores induce a weaker *trans*-influence on the substituent positioned in *trans* position to the multiple bond (a *trans*-effect is described as the lengthening of the bond distance between a metal and some coordinated atom when this atom occupies a position *trans* to the multiple bond). A decreased *trans*-influence allows the formation of an octahedral structure that can accommodate a tripodal ligand in a distorted *fac* configuration. This *fac* arrangement is impossible to be achieved in a distorted, umbrella-shaped square-pyramidal geometry commonly exhibited by Tc-nitrido complexes.

However, despite their favorable coordination topologies capable of almost perfectly nesting a tripodal ligand, there exists a significant difference between the σ frontier orbitals of Tc-trioxo and Tc-tricarbonyl metallic fragments originating from their energy difference. More precisely, the three σ-acceptor orbitals of the Tc-trioxo core lie at lower energies than those of the corresponding orbitals of the Tc-tricarbonyl core. This energy difference suggests that tripodal ligands possessing hard σ-donor atoms (e.g., N, O) are more suitable for binding the hard Tc-trioxo group whereas the soft Tc-tricarbonyl core prefers to bind softer tripodal ligands as, for example, poly(mercaptoazolyl)borates (Figure 1) where one sulfur donor atom is linked to each aromatic ring. However, this tendency should be taken with some caution because poly(pyrazolyl)methanes (Figure 1) form stable complexes also with the *fac*-Tc(CO)_3_ fragment. Based on the very simple model pictured here, it is unclear whether poly(pyrazolyl)methanes should be considered as softer scorpionate ligands or they fail to follow the rough hard-soft rule outlined above. Notwithstanding its simplicity, the qualitative model sketched above correctly predicts the various categories of Tc complexes that will be shortly described in the following sections.

## 4. Tc-Oxo and Tc-Sulfido Scorpionate Complexes

One of the very first examples of Tc(V)-oxo complexes prepared under aqueous conditions and fully characterized was the Tc-oxo complex prepared by reacting excess of the potassium salt of tris(pyrazolyl)borate (KHBpz_3_) with [TcO_4_]^−^ in the presence of KBH_4_ and HCl to yield the complex [Tc(O)(HBpz_3_)Cl_2_] [24]. The molecular structure of this complex was fully determined and revealed that the metallic center lies in a distorted octahedral coordination environment where the six ligating atoms are one oxygen, two chlorines, and the three nitrogen atoms of the HBPz_3_ ligand. As expected, these three nitrogen donors occupy three facial positions of the coordination octahedron. However, only two of the three Tc–N bond lengths fall in the usual range for this type of chemical bond. The Tc–N bond *trans* to the Tc-oxo functionality is approximately. 0.2 Å longer than the normal Tc–N bond length shown by the other two nitrogen atoms that are *trans* to the chlorine ligands. Although the magnitude of this structural *trans*-effect induced by the oxo ligand is measurable, it is not strong enough to elongate the Tc–N bond length until it breaks, thus producing a square-pyramidal geometry. Instead, the Tc-oxo complex is able to accommodate the tripodal ligand and the only consequence of the *trans-*effect is the distortion of the triangular facial configuration and, in turn, of the octahedral geometry. 

The complex, [Tc(O)Cl_2_(HBpz_3_)] was subsequently used for the preparation of the first mononuclear complex containing a terminal [Tc≡S]^3+^ core, which is isolectronic with the [Tc≡O]^3+^ functionality. This approach involved the use of B_2_S_3_ as a source of sulfide ([S]^2−^) groups under strictly anhydrous conditions. More precisely, the first mononuclear Tc(V) complex possessing a terminal Tc≡S bond was prepared by reacting the complex [Tc(O)Cl_2_(HBpz_3_)] with B_2_S_3_, in anhydrous CH_2_CI_2_, to produce the corresponding sulfido complex, [Tc(S)Cl_2_(HBpz_3_)]. In such reaction, the sterically encumbering ligand [HBpz_3_]^−^ stabilizes the overall structure of both the initial and final complexes, thus allowing the oxo group being substituted by the sulfido group. At present, the complex [Tc(S)Cl_2_(HBpz_3_)] still remains the only example of mononuclear Tc(V) complex containing a terminal Tc≡S multiple bond [16]. The Tc-oxo complex [Tc(O)Cl_2_(HBpz_3_)] was also used as precursor for the synthesis of Tc(III) scorpionate derivatives of the type [Tc(L)Cl_2_(HBpz_3_)] (L = PPh_3_, OPPh_3_, pyridine) [25].

## 5. Tc-Trioxo Scorpionate Complexes

**Figure 4:**
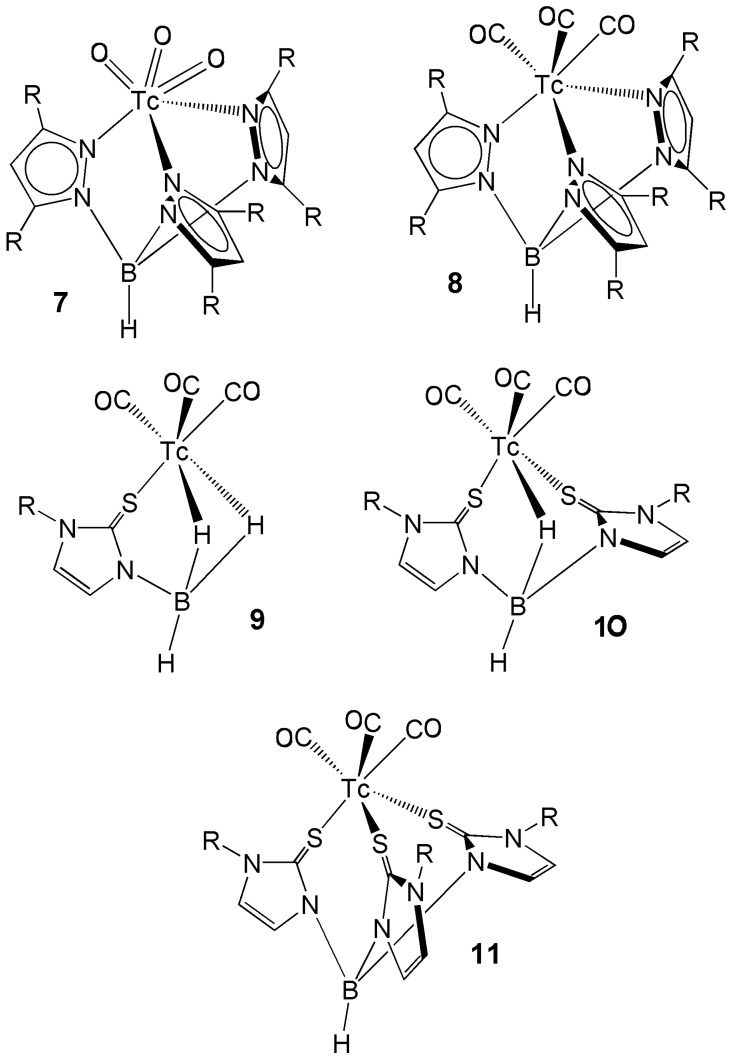
Schematic drawings of the chemical structures of the most important families of technetium scorpionate complexes. [Tc(O_3_)HB(R_2_pz)_3_] (**7**), [Tc(CO)_3_HB(R_2_pz)_3_] (**8**), [Tc(CO)_3_H_3_B(Rmz)] (**9**), [Tc(CO)_3_H_2_B(Rmz)_2_] (**10**), [Tc(CO)_3_HB(Rmz)_3_] (**11**).

In general, stabilization of high oxidation states of technetium can only be attained by using so-called hard scorpionates characterized by σ-donor nitrogen atoms. The Tc-trioxo core ([Tc(O_3_)]^+^) embodies the technetium ion in its highest possible oxidation state (+7) [26] and it exhibits the *C_3v_* scaffold that is particularly well suited for harboring a tripodal ligand. A general drawing of the structure of these scorpionate complexes is presented in Figure 4 [5,9,26,27]. Different synthetic pathways have been investigated for the synthesis of Tc-trioxo scorpionate complexes. One route makes use of [TcO_4_]^−^ as starting material. However, the reaction carried out through the addition of the sodium salt of the scorpionate ligand [HBpz_3_]^−^ to pertechnetate, in a mixture of H_2_SO_4_ and ethanol, to afford the complex [Tc(O_3_)HBpz_3_], is the only reported route of formation of a Tc-trioxo scorpionate complex starting from [TcO_4_]^−^ [28]. This suggests that the Tc-tetraoxo anion exhibits a low reactivity towards tripodal ligands and, thus, some kind of preliminary chemical activation is usually required. For example, addition of BF_3_·OEt_2_ to [TcO_4_]^−^ generates the adduct [Tc(O_3_)(OBF_3_)]^−^, which was subsequently used as precursor for the synthesis of the complexes [Tc(O_3_)HC(pzMe_2_)_3_]^+^ and [Tc(O_3_)HC(pzMe_2_)_3_]^+^ in dry acetone. When benzyl chloride was employed for activation in these reactions, the complexes [Tc(O_3_)(pz_2_CHCOO] and [Tc(O_3_)(pzMe_2_)_2_CHCOO] were isolated, where the Tc-trioxo core is stabilized by the bis(pyrazol-1-yl)acetato ligand and its 3,5-dimethylated derivative [29]. The volatile Tc_2_O_7_ was also used as alternative source of [Tc(O_3_)]^+^ groups through the reaction of the alkali salts of the ligands K[HBpz_3_] and Na[HB(pzMe_2_)_3_] to yield the complexes [Tc(O_3_)HBpz_3_] and [Tc(O_3_)HB(pzMe_2_)_3_] [30].

## 6. Tc-Tricarbonyl Scorpionate Complexes

As expected, investigations of the synthesis of poly(pyrazolyl)borate complexes containing the Tc(I)-tricarbonyl core did not reveal a rich chemical behavior and the preparation of a few complexes such as *fac*-[Tc(CO)_3_HBpz_3_] and *fac*-[Tc(CO)_3_HB(pzMe_2_)_3_] were described using [Tc(CO)_5_Br] or [{Tc(CO)_4_(μ-Cl)}_2_] as starting precursors in organic solvents [8]. The typical coordination arrangement characterizing Tc-tricarbonyl complexes with tripodal hydrotris(pyrazolyl)borate ligands is sketched in Figure 4. Starting from the mixed tricarbonyl-bisphosphane precursor complex, [Tc(CO)_3_(PPh_3_)_2_Cl], the reaction with [HBpz_3_]^−^ produced the complex [Tc(CO)_2_(PPh_3_)HBpz_3_] where one carbonyl group had been removed, thus failing to preserve an intact Tc-tricarbonyl moiety [31].

A far richer chemistry was found after the introduction of thioimidazolyl scorpionates (Figure 1) [6,32,33]. Usually, these ligands are obtained by reacting an alkali metal salt of the borohydride anion [BH_4_]^−^ with the appropriate mercaptoimidazole derivative. Interestingly, the mono-hydro-, di-hydro and tri-hydro derivatives (Figure 1) can be produced by a careful tuning of the reaction conditions (temperature and reagents’ concentrations). Poly(mercaptoimidazolyl)borates are classified as soft scorpionates because they coordinate the metal center through the thione sulfur atom attached to the mercaptoimidazolyl ring. As discussed previously, the *sp*^2^ σ-orbital of the sulfur donor atom overlaps one of the σ-acceptor orbitals of the *fac*-[Tc(CO)_3_]^+^ fragment. However, electron delocalization may occur throughout the ring that functions as a reservoir of electronic density. Possibly, this delocalized π-orbital system may give rise to some interaction with suitable empty π-orbitals on the tricarbonyl metal fragment. This make these soft scorpionates particularly appropriate for stabilizing complexes containing the technetium atom in a low oxidation state as it happens when the *fac*-[Tc(CO)_3_]^+^ metallic fragment is used as chemical synthon [7].

Remarkably, when sodium or lithium salts of tris-, bis- or mono(mercaptoazolyl)borates react with *fac*-[Tc(CO)_3_(H_2_O)_3_]^+^ in aqueous or organic solutions, a variety of complexes containing a variable number of substituted thioimidazolyl rings can be isolated. More precisely, the complexes *fac*-[TcHB(mzR)_3_(CO)_3_], *fac*-[TcH_2_B(mzR)_2_(CO)_3_] and *fac*-[TcH_3_B(mzR)(CO)_3_] (R = Me) were isolated and structurally characterized (Figure 4). It is worthy to note that the complex *fac*-[TcH_3_B(mzMe)(CO)_3_] is the first example of a technetium complex anchored by a mono(azolyl)trihydroborate, which is the lightest scorpionate of the poly(azolyl)borate family [34]. The crystal structures of *fac*-[TcH_2_B(mzMe)_2_(CO)_3_] and *fac*-[TcH_3_B(mzMe)(CO)_3_] consistently revealed the presence of one or two B···H···Tc bridges, respectively. These complexes with mono- and bis-agostic hydride coordination were prepared in aqueous solution by replacing one or two water molecules of the *fac*-[Tc(CO)_3_(H_2_O)_3_]^+^ precursor with agostic hydrogen atoms (*κ^3^-HSS* and *κ^3^-HHS* coordination modes).

In the last years, there was a growing interest for another important family of scorpionates composed by tris(pyrazolyl)alkanes (Figure 1) [35,36]. This interest was mostly fueled by the discovery that a few technetium complexes with these chelators demonstrated attractive properties for diagnostic imaging of myocardial perfusion (see below). These ligands possess a superior stability in aqueous solutions as compared to the analogous tris(pyrazolyl)borates and, because of their preorganized tripodal topology, they are obvious candidates for stabilization of the *fac*-[Tc(CO)_3_]^+^ moiety. A schematic illustration of the chemical structure of these complexes is pictured in Figure 4.

## 7. ^99m^Tc-Scorpionate Radiopharmaceuticals

Despite the extensive and elegant work carried out on the synthesis of technetium complexes with scorpionate ligands using different Tc-cores, the translation of this chemistry to the preparation of diagnostic ^99m^Tc-radiopharmaceuticals remains limited. The main challenge for accomplishing this endeavor commonly arises because of the more stringent conditions that have to be accomplished in the preparation of ^99m^Tc-radiopharmaceuticals. Normally, the preparation of a ^99m^Tc agent should take place in an aqueous environment and in a concentration interval that is far lower than the milliomolar range (10^−3^ M) commonly employed in conventional chemical syntheses. This concentration level typically ranges between 10^−6^–10^−9^ M (conventionally called *no-carried-added* (nca) level). This remarkable drop of reagents’ concentrations may always bring about a dramatic change in the kinetic of the reaction that, ultimately, could fail to afford the same product isolated in macroscopic amounts. Fortunately, the broad variety of technetium oxidation states and chemical structures has always provided efficient synthetic solutions to be exploited for the preparation of ^99m^Tc-radiopharmaceuticals. For instance, in the field of scorpionate technetium chemistry, the development of an efficient route, at nca level and under full aqueous conditions, for the production of the *fac*-[^99m^Tc(CO)_3_(OH_2_)_3_]^+^ synthon has been a key advancement towards the development of this category of organometallic ^99m^Tc-radiopharmaceuticals.

Accordingly, the preparation of the ^99m^Tc-tricarbonyl complexes with tris-, bis- or mono(mercaptoimidazolyl)borates, previously obtained at the macroscopic level, were also carried out at the nca level by reacting *fac*-[^99m^Tc(CO)_3_(OH_2_)_3_]^+^ with the sodium salts of the corresponding scorpionates, at room temperature, to afford the ^99m^Tc-compounds *fac*-[^99m^TcHRB(mzMe)_3_(CO)_3_] (R = H, Ph), *fac*-[^99m^TcH_2_B(mzMe)_2_(CO)_3_] and *fac*-[^99m^TcH_3_B(mzMe)(CO)_3_]. Biodistribution studies in animal models showed that these complexes are able to penetrate the intact blood brain barrier (BBB), presumably because of their neutral charge and lipophilic character [34]. The permeability of BBB to the passage of these complexes was harnessed to develop imaging agents targeting 5-HT1A receptors in the central nervous system. For this purpose, the pharmacophore 2-(methoxyphenyl)piperazine displaying high affinity for 5-HT1A receptors, was appended to one or both mercaptoimidazolyl rings of the dihydro-scorpionate [H_2_B(mzMe)_2_]^−^ (Figure 1) with variable spacers. The resulting bifunctional ligands were then reacted with the *fac*-[^99m^Tc(CO)_3_(OH_2_)_3_]^+^ to yield ^99m^Tc-products bearing pendant mono- and bis-substituted pharmacophores. Biological studies revealed only a negligible brain uptake that prevented their further evaluation as 5-HT1A-receptor imaging agents [37,38].

**Figure 5:**
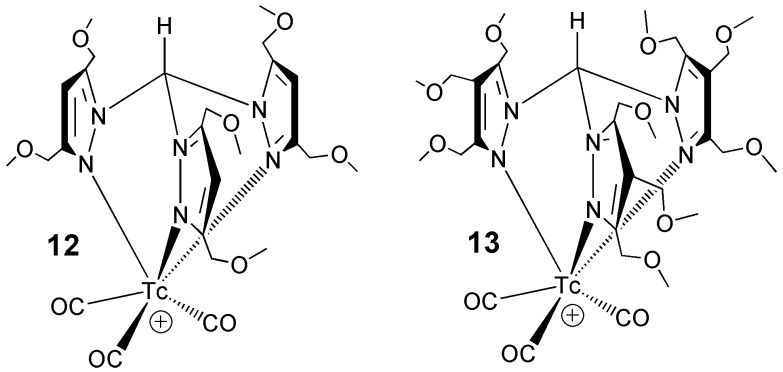
Drawings of the chemical structure of the two novel myocardial ^99m^Tc-tracers, [Tc(CO)_3_HC{(CH_2_OMe)_2_pz}_3_] and [Tc(CO)_3_HC{(CH_2_OMe)_3_pz}_3_] based on scorpionate ligands.

More interesting results have been unearthed through the study of the biological properties of ^99m^Tc-complexes with derivatives of tris(pyrazolyl)methane (Figure 1). The general structure of these complexes is represented in Figure 5. Obviously, the simplest member of this family is the complex [Tc(CO)_3_(HCpz_3_)]^+^ carrying a monopositive charge. This stimulated the further investigation of this compound as potential imaging agent for myocardial perfusion since it was well-established that a number of cationic ^99m^Tc-complexes are selectively accumulated by myocardial cells, thus suggesting that a monopositive charge is a key feature for promoting selective uptake by cardiac tissue. Biodistribution experiments in mice nicely confirmed this expectation and showed that the ^99m^Tc analogue, [^99m^Tc(CO)_3_(HCpz_3_)]^+^, along with other derivatives of the type [^99m^Tc(CO)_3_(RCpz_3_)]^+^ (R = MeOCH_2_, EtOCH_2_, PrOCH_2_), were efficiently extracted by myocardial cells [39].

Actually, the most favorable biological properties were observed with the derivatives pictured in Figure 5, where the scorpionate ligands HC[3,5-(MeOCH_2_)_2_pz]_3_ and HC[3,4,5-(MeOCH_2_)_3_pz]_3_ were employed [40,41,42]. These two ligands bear two or three ether groups on the azolyl ring, respectively. When reacted with the precursor *fac*-[Tc(CO)_3_]^+^, as sodium salts, these ligands led to the formation of the monocationic compounds *fac*-[^99m^Tc(CO)_3_{HC[3,5-(MeOCH_2_)_2_pz]_3_}]^+^ and *fac*-[^99m^Tc(CO)_3_{HC[3,4,5-(CH_3_OCH_2_)_3_pz]_3_}]^+^. Biological results demonstrated that these novel radiotracers have a remarkably high and stable heart uptake combined with a fast blood and liver clearance that are always highly desirable pharmacokinetic properties to improve the quality of diagnostic images. A planar scan of the biodistribution of these tracers in rats confirmed the excellent image resolution of the cardiac region, thus suggesting that these new ^99m^Tc-radiopharmaceuticals could play a potential role as improved imaging agents for the diagnosis of coronary artery disease.

## 8. Conclusions

The tale of the development of technetium scorpionate complexes is an impressive example of how different scientific fields can separately contribute to generate results that could be of tremendous usefulness for society. The production of scorpionate-based ^99m^Tc-radiopharmaceuticals was possible because of the convergent, interdisciplinary contribution of different disciplines, particularly from the field of chemistry. This effort has become reality by combining results from scorpionate chemistry with methods and technologies of radiopharmaceutical chemistry and radionuclide imaging, thus offering another striking example that only through an interdisciplinary approach is realistically possible to address fundamental problems. In turn, this emphasizes the persistent vigor of the research studies on the properties of scorpionate ligands. Although not yet fully evaluated in clinical trials, the novel myocardial imaging agents based on scorpionate ligands are surely of high interest, particularly when viewed in the context of the recent, outstanding advancements of SPECT technology where image sensitivity, spatial resolution and acquisition times have been dramatically improved. It might happen that, to exploit these new technological opportunities, heart imaging agents having a more favorable heart-to-background ratio (in particular, heart-to-liver ratio) could become essential. However, the application of scorpionate chemistry to the search of novel ^99m^Tc imaging agents (possibly, also with other diagnostic radionuclides) is still in its infancy and it is reasonable to expect further, exciting advancements in the near future.
